# The 3-Minute Diagnostic Confusion Assessment Method severity score correlates with the Delirium Rating Scale–Revised–98 and with biomarkers of delirium

**DOI:** 10.1016/j.bjao.2025.100398

**Published:** 2025-04-21

**Authors:** Cameron Rivera, David Kunkel, Maihlee Her, Simran Qureshi, Robert A. Pearce, Robert D. Sanders, Richard Lennertz

**Affiliations:** 1 Department of Neurological Surgery, Miller School of Medicine, University of Miami, Miami, FL, USA; 2 Department of Anesthesiology, University of Wisconsin School of Medicine and Public Health, Madison, WI, USA; 3 University of Sydney, Sydney, NSW, Australia; 4 Department of Anaesthetics, Royal Prince Alfred Hospital, Camperdown, NSW, Australia

**Keywords:** 3D-CAM, biomarker, CAM, delirium, DRS, postoperative, severity, surgery

## Abstract

**Background:**

Several methods are used to measure delirium severity in the postoperative period. Here, we compare severity scores from two common assessment methods: the 3-Minute Diagnostic Confusion Assessment Method (3D-CAM) and the Delirium Rating Scale–Revised–98 (DRS).

**Methods:**

Data were collected as part of an ongoing observational cohort study of perioperative delirium in patients >65 yr old undergoing major elective surgery with an anticipated hospital stay of at least 2 days. Patients were excluded if they had a documented history of dementia, resided in a nursing home, underwent neurosurgery, or could not complete neurocognitive testing. Patients underwent paired 3D-CAM and DRS assessments before and after operation along with EEG, cognitive testing, and plasma biomarker analysis.

**Results:**

Of 226 subjects enrolled, 204 completed both the 3D-CAM and DRS assessments. Peak 3D-CAM severity (3D-CAM-S) scores correlated with peak DRS severity scores, for both the raw (ρ=0.74, *P*<0.001) and short form method (ρ=0.66, *P*<0.001). Peak 3D-CAM-S raw scores also correlated with delirium duration and severity duration area under the curve measures (ρ=0.71, *P*<0.001 and ρ=0.91, *P*<0.001, respectively). Similar to prior reports with the DRS, 3D-CAM-S raw scores correlated with worse performance on the Trail Making Test B (ρ=0.37, *P*<0.001, *n*=177), slow-wave electroencephalogram power (ρ=0.3, *P*=0.001, *n*=73), and plasma neurofilament light (ρ=0.26, *P*<0.05, *n*=61) and tau (ρ=0.41, *P*<0.001, *n*=63).

**Conclusions:**

The 3D-CAM-S severity scores correlated with DRS, delirium duration, and biomarkers of delirium. The 3D-CAM, which is easier to implement than the DRS in postoperative patients, may provide a comparable assessment of delirium severity in this population.

**Clinical trial registration:**

NCT03124303.

Postoperative delirium affects 10–50% of geriatric patients and impacts the incidence of adverse events, hospital length of stay, and hospital cost.^1^, ^2^, ^3^ Clinical instruments are often used to assess the incidence and severity of delirium through questions, activities, and observations. Severity scores help to identify subsyndromal symptoms or measure the severity of disease. However, several methods for assessing delirium exist and it remains unclear which method best captures delirium severity under postoperative conditions.

Here, we focus on severity scores derived from two common methods of assessing delirium: the 3-Minute Diagnostic Confusion Assessment Method (3D-CAM)^4^ and the Delirium Rating Scale–Revised–98 (DRS).^5^ The 3D-CAM was developed as a means to quickly and reliably diagnose delirium, and the DRS to both diagnose delirium and to quantify its severity. The 3D-CAM gained popularity because of its ease of use; it takes an average of 3 min and was designed for administration by trained non-clinicians. However, the 3D-CAM did not initially provide an assessment of delirium severity. To address this shortcoming, two methods were subsequently developed to provide clinicians and researchers with severity scores directly from the 3D-CAM (3D-CAM-S). The ‘short form’ method (3D-CAM-S short form) derives a severity score ranging from 0 to 7 based on the presence and severity of four features of delirium,^6^ similar to the Confusion Assessment Method short form severity score.^7^ Alternatively, the ‘raw’ method (3D-CAM-S raw) utilises the sum of positive items from the 3D-CAM to create a severity score ranging from 0 to 20.^8^ It has been suggested that the expanded dynamic range of the raw method may improve associations with biomarkers of delirium.^9^

Although severity scores can be calculated from either the 3D-CAM or DRS, there remain inherent differences between the assessments. The 3D-CAM does not directly evaluate some features of delirium, such as sleep or visuospatial impairment; other features, such as psychological disturbances, are scored as either ‘present’ or ‘absent’ on the 3D-CAM, but receive graded scores on the DRS. Both assessments have shown high sensitivity and specificity for diagnosing delirium,^4^^,^^5^ and yet these differences may be important when measuring delirium severity. Indeed, we expect the DRS to correlate more strongly with biomarkers because it assesses additional features of delirium. However, others have used item response theory to demonstrate that several different methods measure the same underlying construct of delirium severity and have developed crosswalks between some instruments.^10^ Currently, a crosswalk is not available for the 3D-CAM.

It is also unclear whether peak severity remains the best measure of disease burden. While several studies associated higher delirium severity with worse clinical outcomes,^9^^,^^11^^,^^12^ others report similar findings using delirium duration.^13^ A combined measure of delirium severity and duration (area under the curve, AUC) may capture the postoperative course of delirium even more accurately. Relatively few studies consider both severity and duration,^14^^,^^15^ and their relationship deserves further consideration as researchers investigate the clinical implications and pathophysiology of delirium.

In this study, we compare different measures of delirium severity in a single cohort of postoperative patients. First, we consider the relationship and agreement between the peak severity scores, delirium length, and combined delirium-severity AUC. Then, we consider the association of these measures with biomarkers that have previously been associated with delirium using the DRS.^16^, ^17^, ^18^, ^19^, ^20^ Here, observed agreement increases our confidence in comparing results across studies that have used different severity measures.

## Methods

### Study design

We performed an exploratory analysis of data collected in an ongoing observational cohort study of postoperative delirium (IPOD-B3). The study was registered with ClinicalTrials.gov (NCT03124303) and approved by the University of Wisconsin-Madison Institutional Review Board (2015-0374). Consent was obtained at a baseline visit in accordance with the Declaration of Helsinki. Data are reported in accordance with the Strengthening the Reporting of Observational Studies in Epidemiology (STROBE) guidelines. Our primary objective was to compare delirium severity scores derived from the DRS and 3D-CAM assessment methods in a postoperative patient population. Exploratory objectives were 1) to compare the ‘raw’ and ‘short form’ scoring methods for the 3D-CAM, 2) to assess the correlation of peak severity scores to other measures of delirium, and 3) to compare severity measures against cognitive function, electrophysiological features, and biological markers of delirium. We have previously reported associations between peak DRS and these measures.^16^, ^17^, ^18^, ^19^, ^20^

### Recruitment

Patients >65 yr old undergoing major elective surgery with an anticipated hospital stay of at least 2 days were recruited to participate. Participants were excluded if they had a documented history of dementia, resided in a nursing home, or could not complete neurocognitive testing. Neurosurgical procedures were excluded. Our primary analysis correlates DRS and 3D-CAM-S assessments. Therefore, participants missing paired DRS and 3D-CAM assessments at the peak DRS time point were excluded from analysis. Participants with missing electrophysiological or biological data were excluded from secondary analyses.

### Delirium assessments, cognitive testing, biomarker collection, and EEG recordings

Participants completed a preoperative baseline assessment and postoperative assessments twice daily for up to 4 days (postoperative days, PODs) or until delirium resolved, whichever was longer. Delirium incidence was assessed via the 3D-CAM or Confusion Assessment Method – ICU (CAM-ICU). The CAM-ICU was only used to determine the incidence of delirium when neither the 3D-CAM nor the DRS could be assessed and was not used to assess delirium severity. Delirium severity was assessed with the DRS. DRS assessments with more than two missing items were marked as incomplete. The Trail Making Test B (TMTB) was recorded at the earliest postoperative assessment possible. Plasma samples were collected at baseline and PODs 1–4 in EDTA-containing tubes and centrifuged before being stored at −80°C. Samples were sent for cytokine multiplex assay for interleukin 8 (IL-8) (Eve Technologies, Montreal, QC, Canada). Samples were sent to the University of Gothenburg for analysis of total plasma tau and neurofilament light chain (NfL) as previously described.^21^ High-density EEG data were collected at baseline and after operation using a 256-channel system (Electrical Geodesics, Inc., Eugene, OR, USA).

### Statistical analysis

3D-CAM-S scores were calculated *post hoc* from the 3D-CAM using the short form method and the raw method.^7^^,^^8^ Delirium duration and AUC calculations assume evenly spaced (12-h) intervals between postoperative assessments. Delirium duration was calculated from the number of postoperative delirium or coma (Richmond Aggression Sedation Scale score of −4 or −5) assessments, where each delirium assessment quantified as 12 h of delirium. These did not need to be contiguous, as delirium fluctuated for some participants. AUC was calculated as the product of delirium severity and time measured in number of assessments from POD1 to POD4. If one or two consecutive time points were missing, delirium severity scores were imputed as a mean of the surrounding points. Patients missing their first two POD assessments, or more than two consecutive POD assessments were excluded. TMTB scores were recorded with a maximum time cut-off of 300 s. Biomarker data were not normally distributed. The data were transformed to a log_10_ scale, and the postoperative change was calculated (log_10_[POD1AM] - log_10_[baseline]). Biomarker changes were excluded above a Cooks distance of 5, which resulted in the removal of a single NfL sample. EEG slow wave (0.5–6 Hz) power was calculated as previously described,^17^ and is reported as the postoperative change in power. Biomarkers were compared with peak delirium severity scores, which did not necessarily correspond to the same time point as when biomarkers where collected.

Data were stored using the online database server REDCap.^22^^,^^23^ Spearman correlation was used to measure the strength of association between variables. To compare correlation coefficients between dependent variables, bootstrap resampling of the dataset was performed 1000 times to estimate 95% confidence intervals (CI) for difference between coefficients.^24^ The Akaike Information Criterion (AIC) was calculated as previously described.^25^^,^^26^ Linear regression modelling was used to predict DRS scores from 3D-CAM-S scores. Models with linear and exponentiated terms were compared using AIC and analysis of variance. Simple linear models performed well and were chosen to facilitate interpretation. Predicted DRS scores were then compared with the actual paired DRS scores and intraclass correlation coefficients were calculated using two-way fixed effects and mixed effects models.^27^ Statistical analyses were carried out, and graphs produced, using R (Version 1.2.5033). The data that support the findings of this study and proprietary R codes used to calculate findings are available from the corresponding author, upon reasonable request and with institutional approval.

## Results

**Figure 1:**
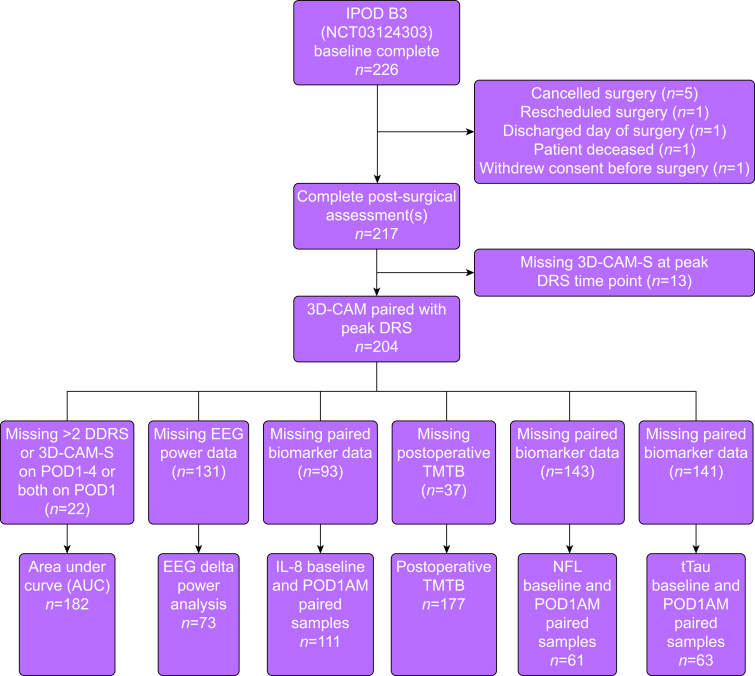
STROBE diagram. 3D-CAM-S, 3-Minute Diagnostic Confusion Assessment Method severity; DRS, Delirium Rating Scale–Revised–98; IL-8, interleukin-8; NfL, neurofilament light chain; POD, postoperative day; STROBE, Strengthening the Reporting of Observational Studies in Epidemiology; TMTB, Trail Making Test B.

Of 226 participants enrolled, 204 underwent surgery and completed paired 3D-CAM and DRS assessments at the time of their peak DRS assessment (Fig. 1). Some 27.5% (56/204) of participants were diagnosed with delirium during their postoperative recovery according to 3D-CAM or CAM-ICU assessments. Subject characteristics are reported in Table 1. Subjects were excluded from secondary analyses because of missing data, as outlined in Figure 1. Differences in the characteristics between patients with and without delirium in this cohort have been previously reported.^16^, ^17^, ^18^, ^19^, ^20^

**Table 1:** Participant characteristics. Patients were classified with delirium if any postoperative CAM assessment was positive for delirium. Blood loss was missing for five cardiac/thoracic/vascular patients without delirium and three cardiac/thoracic/vascular patients with delirium. BMI, body mass index; IQR, inter-quartile range; NSQIP-D, National Surgical Quality Improvement Program Risk Calculator – Death.

	Delirium				No delirium			
	*n*	Median	IQR	Range	*n*	Median	IQR	Range
Age (yr)	56	71	69–75	65–83	148	72	68–75	65–85
Sex (%)								
Female	25	(12)			52	(25)		
Male	31	(15)			96	(47)		
BMI (kg m^−2^)		28.9	24–32	13–50		28.6	25–31	18–47
Education (%)								
<12 yr	15	(7)			35	(17)		
12 yr	2	(1)			2	(1)		
>12 yr	37	(18)			111	(55)		
NSQIP-D		3	0.9–6.1	0.1–26.6		0.9	0.3–2.7	0–25.5
Operating time (min)		396	269–530	125–938		259	187–350	69–596
Blood loss (ml)								
Cardiac/thoracic/vascular	30	700	200–6450	0–31 000	66	150	50–475	0–7000
Other	23	650	350–1200	25–4500	77	300	100–600	0–3000

### 3D-CAM-S scores correlate with the DRS

**Figure 2:**
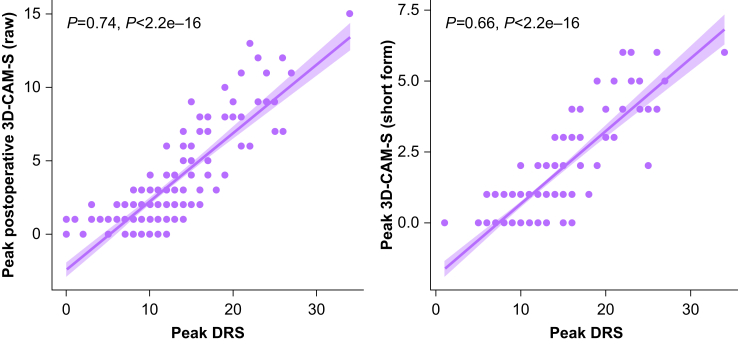
Correlations between peak 3D-CAM-S and peak DRS (left, ρ=0.74, *P*<0.001, *n*=204) and between peak 3D-CAM-S short form and peak DRS (right, ρ=0.66, *P*<0.001, *n*=204). 3D-CAM, 3-Minute Diagnostic Confusion Assessment Method; DRS, Delirium Rating Scale–Revised–98.

As we have previously reported associations with respect to peak delirium severity scores, here we focused on comparisons between peak severity scores. There was a correlation between peak 3D-CAM-S raw and DRS scores (ρ=0.74, *P*<0.001, *n*=204; Fig. 2, left), and between peak 3D-CAM-S short form and DRS scores (ρ=0.66, *P*<0.001, *n*=204; Fig. 2, right). The raw method correlated more strongly with the DRS than the short form method (ρ difference 0.09 [0.02–0.18]). As a sensitivity analysis, peak severity scores for patients with and without delirium were analysed separately. Correlations with the DRS were stronger among patients with delirium for both the raw method (ρ_*delirium*_ *-* ρ_*no-delirium*_ 0.30 [0.13–0.47]) and the short form method (ρ_*delirium*_ *-* ρ_*no-delirium*_ 0.46 [0.27–0.64]). Among patients with delirium, the raw method and short form methods correlated similarly with the DRS (ρ difference 0.01 [−0.10 to 0.11]). Similar to peak scores, paired DRS and 3D-CAM-S raw severity scores were also correlated (ρ=0.66 [0.62–0.69]) and correlations were stronger among patients with delirium (ρ_*delirium*_ *-* ρ_*no-delirium*_ 0.21 [0.07–0.27]).

We also considered the DRS, 3D-CAM-S raw, and 3D-CAM-S short form as three different raters of delirium severity and normalised their scales using simple linear regression. Peak DRS scores and predicted peak DRS scores from the raw (7.8 + 1.37x) and short form (8.8 + 2.66x) methods were compared using intraclass correlation statistics. For both two-way random effects and two-way mixed effects models, the correlation coefficient demonstrated ‘good’ agreement between the methods (0.86 [0.83–0.89]).^27^

To further illustrate the relationship between DRS and 3D-CAM, we predicted peak DRS scores from peak 3D-CAM-S raw scores using a linear mixed effects model. We found that 55% of scores fell within 2 points, and 95% within 5 points, of the actual paired DRS score. This can also be seen in the spread of DRS scores associated with a particular 3D-CAM-S raw score in Figure 2. For all paired assessments, the spread was slightly wider; 53% of scores fell within 2 points and 95% within 7 points of the actual paired DRS score.

### Peak delirium severity scores correlate with delirium duration and AUC measures

**Figure 3:**
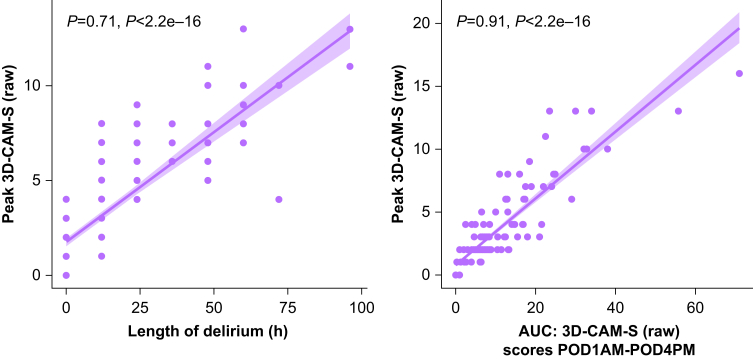
Correlations between delirium length and peak DRS (left, ρ=0.71, *P*<0.001, *n*=182) and between delirium severity and duration AUC and peak delirium severity (right; ρ=0.9, *P*<0.001, *n*=182). 3D-CAM, 3-Minute Diagnostic Confusion Assessment Method; AUC, area under the curve; DRS, Delirium Rating Scale–Revised–98; POD, postoperative day.

The duration of delirium, calculated from the total number of assessments showing delirium, correlated with peak severity scores on the 3D-CAM-S raw (ρ=0.71, *P*<0.001; Fig. 3, left). Delirium AUC was calculated from severity scores over PODs 1–4. Twelve participants were discharged before POD 4 and 31 participants had delirium beyond day 4. AUC scores demonstrated a direct correlation with peak severity scores on the 3D-CAM-S raw (ρ=0.91, *P*<0.001; Fig. 3, right). Similar relationships were noted for the DRS and 3D-CAM-S short form (data not shown).

### Severity measures correlate with biomarkers of delirium

**Figure 4:**
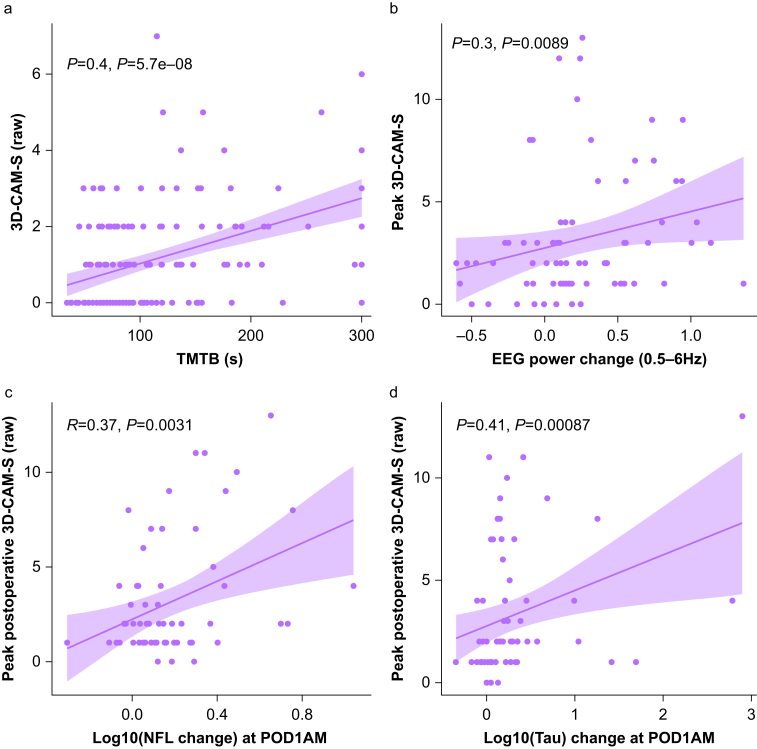
Correlations between peak 3D-CAM-S and biomarkers of delirium: (a) TMTB (ρ=0.4, *p*<0.001, *n*=177), (b) EEG delta power change from preop (ρ=0.3, *P*=0.009, *n*=73), (c) NfL (log change from baseline; ρ=0.37, *P*<0.003, *n*=61), (d) tau (log change from baseline; ρ=0.41, *P*<0.003, *n*=63). 3D-CAM-S, 3-Minute Diagnostic Confusion Assessment Method severity; TMTB, Trail Maker Test B; POD, postoperative day.

First, we examined the relationship between 3D-CAM-S raw scores and features of delirium that have previously correlated with the DRS. Paired 3D-CAM-S raw scores inversely correlated with performance on the postoperative TMTB, a measure of executive function and visual attention (ρ=0.37, *P*<0.001, *n*=177; Fig. 4a),^16^ and peak 3D-CAM-S raw scores directly correlated with increases in slow-wave EEG power (ρ=0.31, *P*=0.01, *n*=73; Fig. 4b).^17^ Peak 3D-CAM-S raw scores also correlated with increases in plasma concentrations of proteins associated with neuronal injury: NfL (ρ=0.26, *P*<0.05, *n*=61; Fig. 4c) and total tau (ρ=0.41, *P*<0.001, *n*=63; Fig. 4d).^18^, ^19^, ^20^

Next, we examined how different severity measures correlate to cognitive function, electrophysiological features, and biological markers of delirium. Table 2 lists the correlation coefficient with estimated 95% CI for each severity measure. Generally, correlations were similar among the different severity measures, with ρ values falling within the 95% CI of each other and 95% CIs that do not include 0. Correlations between delirium AUC and the biomarkers NfL and total tau were weaker and had 95% CI that included 0, indicating that these associations would not be considered significant by formal hypothesis testing. We also calculated AIC between the different measures of delirium severity and each biological marker. No measure of delirium severity consistently or convincingly fit the biological data better than any other (Supplementary Table S1). Of note, delirium AUC demonstrated the worst fit for both the NfL and tau data.

**Table 2:** Correlation of different measures of severity with biomarkers of delirium. Rho values with bootstrapped 95% confidence intervals are reported. EEG slow wave (0.5–6 Hz) power reported as change from baseline. NfL and tau values are log-transformed change from baseline. 3D-CAM, 3-Minute Diagnostic Confusion Assessment Method; AUC, area under the curve; DRS, the Delirium Rating Scale–Revised–98; NfL, neurofilament light chain; TMTB, trail maker test B.

	TMTB		EEG SW power		NfL		Tau	
Peak DRS	0.55	[0.43–0.66]	0.35	[0.12–0.56]	0.31	[0.05–0.53]	0.34	[0.11–0.54]
Peak 3D-CAM-S (raw)	0.55	[0.42–0.66]	0.30	[0.10–0.49]	0.25	[0.02–0.48]	0.40	[0.19–0.60]
Peak 3D-CAM-S (short form)	0.36	[0.22–0.50]	0.29	[0.07–0.49]	0.38	[0.14–0.62]	0.26	[0.01–0.48]
Length	0.44	[0.31–0.55]	0.36	[0.17–0.55]	0.37	[0.12–0.59]	0.30	[0.08–0.51]
AUC	0.52	[0.40–0.64]	0.37	[0.14–0.56]	0.10	[−0.20 to 0.40]	0.22	[−0.05 to 0.56]

## Discussion

In this cohort, we found that the DRS, 3D-CAM raw, and 3D-CAM short form methods yielded similar severity scores in postoperative patients, demonstrated by strong correlation and agreement. There was a stronger correlation between the scores for patients with than without delirium. Yet, the two severity scores were not ‘interchangeable’; many scores predicted from the 3D-CAM-S raw differed by more than 2 points from the actual paired DRS score. For example, a participant with a 3D-CAM-S raw score of 3 may score between 9 and 13 on the DRS, but only about half of the time. This may be because the DRS assesses features of delirium that are not assessed by the 3D-CAM and provides a more detailed assessment of features that are shared by both. This gives the DRS a wider dynamic range than the 3D-CAM-S which may offer advantages in assessing subsyndromal delirium or individual features of delirium. However, the wider dynamic range of the DRS did not lead to stronger correlations with biomarkers. The DRS also takes longer to administer and more expertise to score, disadvantages which motivated the development of the 3D-CAM from the Confusion Assessment Method.^4^^,^^28^ In our experience, patients have difficulty completing the DRS after surgery, especially the visuospatial assessments where there are often limitations in using a pen because of pain, tubing connected to indwelling catheters, etc.

Several different measures have been used to quantify delirium severity. Peak severity scores have been widely used in postoperative patients when assessing biomarkers and postoperative outcomes. Delirium duration has been used as an outcome measure for delirium interventions^13^ and has been associated with outcomes such as length of stay.^29^ Here, we found that peak severity scores correlated with duration and AUC measures. Importantly, the DRS, 3D-CAM, and duration measures all agreed in their comparisons against cognitive function, electrophysiological features, and biological markers of delirium. In other words, in our study, one would have observed similar results regardless of the severity measure used.

Our study has limitations. We cannot exclude the possibility of confounding, though this seems unlikely to affect comparisons between delirium assessments in the same participant, particularly when the same investigator conducted both paired assessments. Delirium and its severity fluctuate, and this could have occurred during the collection of our paired assessments, leading to additional variation and decreasing the strength of correlation in our analyses. The measurement of delirium duration was limited by the twice daily frequency of our assessment. Some participants were discharged before POD 4 and others had delirium beyond POD 4, adding variability to our AUC severity calculation. This may have contributed to differences in correlations between the AUC measure and biomarkers of delirium. We did not have EEG and plasma biomarker data for all participants, limiting the sample size available for these comparisons. Item response theory has been used to compare assessment methods and develop crosswalks between them.^10^ Here, we assessed the strength of correlation between assessments because this allowed us to compare different measures of severity and their relationship with biological markers. We only report estimated CI for the latter comparisons, as the comparisons seem too broad to merit formal hypothesis testing but are of interest to the field.

## Conclusion

In summary, the 3D-CAM provides a comparable assessment of delirium severity and, in our opinion, is easier to implement than the DRS in postoperative patients. Their agreement is not perfect, as any given 3D-CAM score may correspond to a range of DRS scores in paired assessments. All severity scores correlated with biomarkers of delirium, with peak severity scores performing similarly to delirium duration. Nevertheless, research objectives and logistical considerations, such as assessor experience and patient burden, must be weighted when choosing which assessment method to use.

## Authors’ contributions

Study design: RDS, RAP, RL

Data acquisition: DK, CR, SQ, MH

Data analysis and interpretation: CR, RL

Drafting of the manuscript: CR, RDS, RL

Critical revision and final approval: all authors

## Funding

The National Institute of Health (R01AG063849, 2R01AG063849).

## Declarations of interest

The authors declare that they have no conflicts of interest.
