# Supplementary material for: The 3-Minute Diagnostic Confusion Assessment Method severity score correlates with the Delirium Rating Scale–Revised–98 and with biomarkers of delirium
Source: BJA Open. 2025 Apr 21;14:100398. doi: 10.1016/j.bjao.2025.100398 (PMC12051057; doi:10.1016/j.bjao.2025.100398)
Supplement: Multimedia component 1 [file mmc1.docx]

|  | **TMTB** | | **EEG SW Power** | | **NfL** | | **Tau** | |
| --- | --- | --- | --- | --- | --- | --- | --- | --- |
| **Peak DRS** | **1700** | **0.95** | 71 | 0.06 | -24 | 0.21 | 105 | 0.09 |
| **Peak 3D-CAM-S (raw)** | 1706 | 0.05 | 70 | 0.10 | -23 | 0.17 | **102** | **0.43** |
| **Peak 3D-CAM-S (short f.)** | 1717 | 0 | 72 | 0.04 | -24 | 0.25 | 104 | 0.12 |
| **Length** | 1739 | 0 | 71 | 0.05 | **-25** | **0.32** | 102 | 0.32 |
| **AUC** | 1718 | 0 | **66** | **0.75** | -20 | 0.05 | 106 | 0.05 |

Supplemental Table 1: Comparisons of model fit. AIC (*left*) and AIC weight (*right*) were calculated to compare linear regression models of the different severity measures against each biomarker. For example, column “TMTB” compares how well each severity measure fits the postoperative TMTB data. Comparisons are only relevant within a column. The lowest AIC value for each column is noted in **bold** font.
